# Progression on the Roles and Mechanisms of Tumor-Infiltrating T Lymphocytes in Patients With Hepatocellular Carcinoma

**DOI:** 10.3389/fimmu.2021.729705

**Published:** 2021-09-03

**Authors:** Xiaoqin Zheng, Wenjie Jin, Shanshan Wang, Huiguo Ding

**Affiliations:** ^1^Department of Gastrointestinal and Hepatology, Beijing You’An Hospital, Capital Medical University, Beijing, China; ^2^Institute for Research in Biomedicine, Università della Svizzera italiana, Bellinzona, Switzerland; ^3^Institute of Microbiology, Eidgenössische Technische Hochschule (ETH) Zürich, Zurich, Switzerland; ^4^Beijing Institute of Hepatology, Beijing You’An Hospital, Capital Medical University, Beijing, China

**Keywords:** hepatocellular carcinoma, tumor-infiltrating lymphocyte, T lymphocyte, interaction, signaling pathway

## Abstract

Primary liver cancer (PLC) is one of the most common malignancies in China, where it ranks second in mortality and fifth in morbidity. Currently, liver transplantation, hepatic tumor resection, radiofrequency ablation, and molecular-targeted agents are the major treatments for hepatocellular carcinoma (HCC). Overall, HCC has a poor survival rate and a high recurrence rate. Tumor-infiltrating lymphocytes (TILs) have been discovered to play essential roles in the development, prognosis, and immunotherapy treatment of HCC. As the major component cells of TILs, T cells are also proved to show antitumor and protumor effects in HCC. Foxp3+, CD8+, CD3+, and CD4+ T lymphocytes are the broadly studied subgroups of TILs. This article reviews the roles and mechanisms of different tumor-infiltrating T lymphocyte subtypes in HCC.

## Introduction

According to the International Agency of Research on Cancer of World Health Organization report, primary liver cancer (PLC) posed a severe threat to people’s life and health, with an estimated 906,000 new cases and 830,000 deaths globally in 2020 (1). In China, liver cancer ranked second in mortality and fifth in morbidity (2). There are three main pathological types of PLC, including hepatocellular carcinoma (HCC), intrahepatic cholangiocarcinoma (CC), and combined HCC/CC. They vary in pathogenesis, biological behavior, histological morphology, treatment, and prognosis. HCC accounts for 85%-90% among them (3). In this article, liver cancer as follows refers to HCC. For HCC, currently available therapeutic options include liver transplantation, tumor resection, radiofrequency ablation, and molecular-targeted agents. However, the survival rate of HCC is low, and the recurrence rate is high, due to the fact that when patients are first diagnosed with HCC, they often have intermediate-stage or advanced-stage HCC, or micro-metastasis tumors (4). The 1-year median survival rate of patients with end-stage HCC was 11% (3), whereas the 1-year recurrence rate following surgical resection was 70% (5), suggesting that these treatment options for HCC were limited. As a result, novel therapies for HCC are still needed. In the past few years, tumor immunotherapy has emerged as a new therapeutic procedure for HCC. Tumor immunotherapy works by generating and strengthening patients’ existing tumor-specific immune responses, which have high efficiency and strong selectivity when compared to traditional tumor therapies. The immunotherapy regimen that each patient should follow is determined by the host’s immunological specificity. In addition, the quantities and distributions of tumor-infiltrating immune cells also assist us in predicting the response of each patient to immunotherapy prior to treatment. The compositions of immune cells, on the other hand, significantly differ between HCC patients and healthy populations (6). A sufficient understanding of the tumor microenvironment (TME) is the key to successful immunotherapy. TME for HCC is a dynamic system that enriches multiple immune cells, such as regulatory T cells (Tregs), tissue-resident memory CD8+ T (Trm) cells, resident natural killer (NK) cells, tumor-associated macrophages (TAMs) (7). Through interacting with each other and the surrounding stromal cells, they form a complex network and play essential roles in the proliferation, migration, invasion, and angiogenesis of HCC (8).

Tumor-infiltrating lymphocytes (TILs) were formerly thought to host immune responses to tumors. With extensive study in this subject, people discover that TILs consist of a variety of lymphocyte subgroups, including innate immune and adaptive immune cells, such as mast cells, macrophages, NK cells, and T lymphocytes. There are many specific antigens on the surfaces of TILs, for example, Foxp3, CD3, CD4, CD8, CD16, CD20, CD56, CD57, CD68, and CD169 (9). It is widely thought that Foxp3, CD3, CD4, and CD8 are linked with T lymphocytes, CD16 with monocytes, CD20 with B lymphocytes, CD56 and CD57 with NK cells, as well as CD68 and CD169 with macrophages. TILs play significant roles in the development, treatment, and prognosis of HCC. Besides, the antitumor or protumor effects of TILs are related to the component of lymphocyte subsets in TME (10). T lymphocytes are the primary cells of TILs in HCC (11). In addition, Foxp3+, CD3+, CD4+, and CD8+ T lymphocytes are the broadly discussed subgroups of TILs (12). However, the results of TILs in HCC remain controversial. Here, the roles and mechanisms of common tumor-infiltrating T lymphocyte subsets in HCC are reviewed.

## Foxp3+ T Lymphocytes

Forkhead box protein P3 (Foxp3), consisting of 12 exons, belongs to the forkhead family of transcriptional factors and regulates the expression of multiple genes. Foxp3 was revealed to be a reliable marker of Tregs as well as a key regulator of their growth and function (13, 14). Tregs were usually divided into two different subgroups: “natural Treg” cells (nTregs) and “induced Treg” cells (iTregs). It was commonly thought that nTregs differentiated from CD4+ CD25+ Foxp3+ T cells in the thymus, whereas iTregs developed from naive CD4+ Foxp3- T cells in the peripheral tissues, such as lymph node and spleen, and universally gained Foxp3 expression outside of the thymus after antigenic stimulation (15). Both two types of Tregs shared the common ability to suppress immune responses, but they used distinct ways to do so. nTregs played roles through cell contact-dependent ways *via* membrane-bound molecules, yet iTregs exerted their functions through cell contact independent ways *via* cytokines including interleukin (IL)-10 and transforming growth factor-β (TGF-β) (16). Now, there’s no reliable biomarker for distinguishing nTregs from iTregs. In recent years, Foxp3+ T cells have been found in many tumors, such as HCC (17), pancreatic ductal adenocarc (18), colorectal cancer (19), gastric cancer (20), and esophageal cancer (21), suggesting that Foxp3+ T cells participated in the tumor progression. However, the subsets of infiltrating Foxp3+ T cells in tumors remained unclear. With immunohistochemical staining analysis, Wang et al. (22) found that Foxp3 was detected in 48% of HCC tumor tissue but not in healthy liver tissue and para-tumor tissue, indicating that the recruitment of Foxp3+ T cells played an important effect in the development of HCC. Foxp3 was mainly present in the nucleus and cytoplasm of tumor cells (22). A synthetic peptide named P60 could bind to Foxp3, restrict its nuclear translocation, and reverse the biological activity for HCC (23), which indicated that Foxp3 was required to enter the nucleus to display its function. However, the expression and regulation mechanisms of Foxp3 are unknown. In tumor cells from patients with HBV-associated HCC, PreS2 overexpression could trigger the Foxp3 overexpression. In contrast, PreS2 knockdown could significantly reduce the expression of Foxp3. This was the first time a direct conclusion that PreS2 triggered Foxp3 expression in HCC was reached (24).

**Figure 1:**
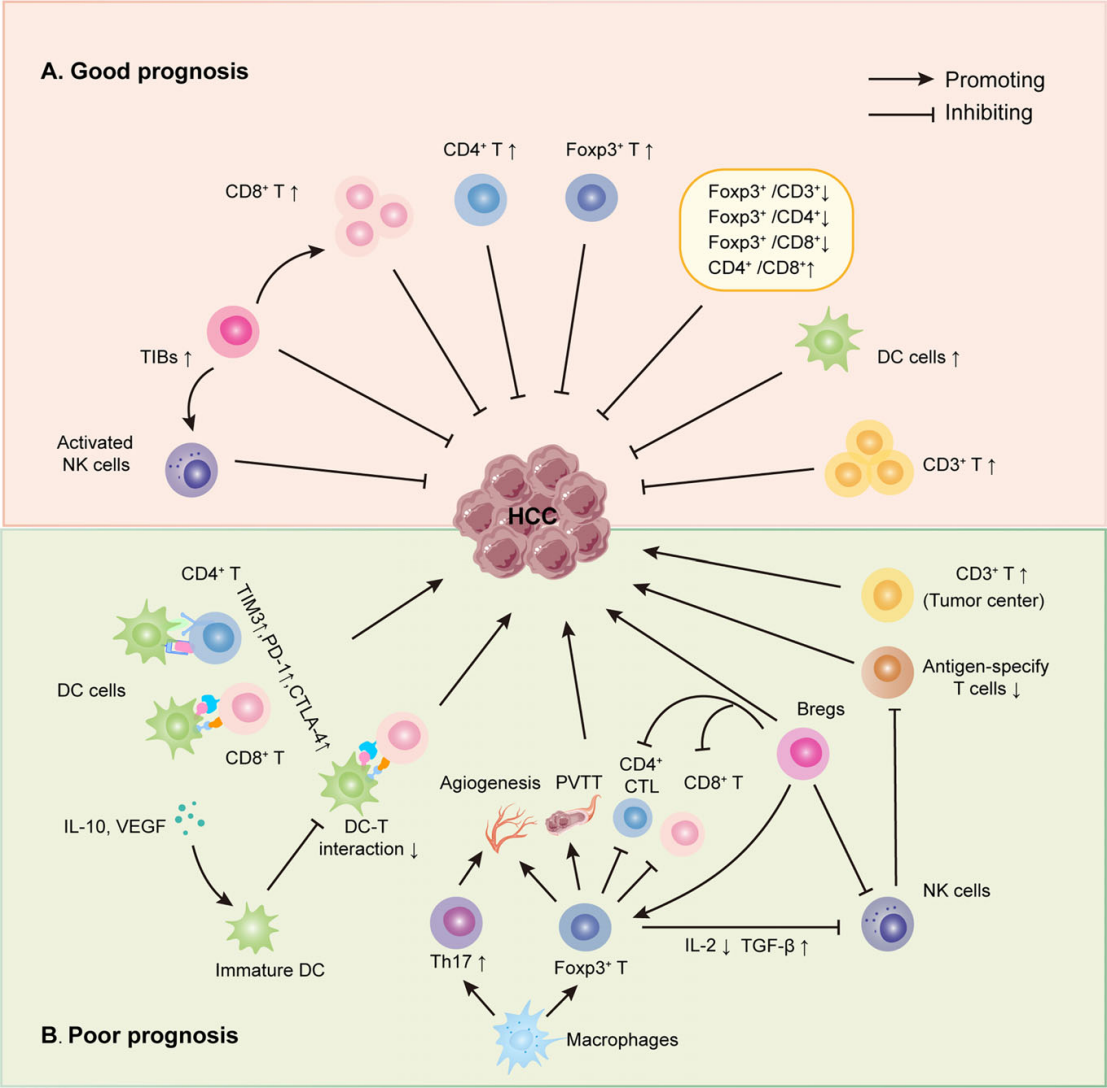
Roles of infiltrating immune cells in HCC. **(A)** The suppressing role of TILs on HCC. Initially, several infiltrating T lymphocytes, including CD3+, CD8+, activated NKs and Foxp3+ TILs, have been discovered to inhibit HCC directly. Secondly, some TILs could interact with other immune cells to suppress HCC, such as TIBs prohibited HCC by increasing CD8+ T cells and activating NK cells. Last, different TIL ratios also were proved to hinder HCC progress, just as low Foxp3+/CD3+ TIL ratio, high CD4+/CD8+ TIL ratio, et al. **(B)** The promoting role of TILs on HCC. Some infiltrating T lymphocytes have been shown to promote HCC directly, like Foxp3+, CD3+ TILs. Also, infiltrating T lymphocytes could interact with other immune cells to boost HCC progress *via* many mechanisms, including reduced DC-T interaction, weakening the function of CD4+ and CD8+ TILs et al.

According to several studies, nTregs promoted tumor growth by limiting antitumor immune responses. iTregs, on the other hand, were shown to favor cancer progression by sustaining peripheral tolerance and controlling cancer-related inflammation (25). The exact roles of nTregs and iTregs in cancer are yet unknown. For this reason, we only talk about total Foxp3+ T cells in this review. Currently, the role of Foxp3+ T cells for HCC prognosis remains disputable. The majority of studies showed that Foxp3+ T cells were related to HCC migration, and high Foxp3 expression predicted a poor prognosis for HCC (17, 26–32) (**Figure 1**). A meta-analysis on HCC, which was conducted by Huang et al. (17), demonstrated that the high Foxp3+ T cells invading group had worse 1, 3, and 5-year survival than the low Foxp3+ T cells infiltrating group. At the same time, the high Foxp3+ T cells infiltrating group had better 1, 3, and 5 year-recurrence than the low Foxp3+ T cells infiltrating group. According to the above conclusion, high Foxp3+ T cells infiltrating was a poor prognostic factor for HCC. The mechanisms in which Foxp3+ T cells promoted HCC progression might include fostering angiogenesis (27), decreasing CD8+ T cells (32), progressively reducing CD4+ CTLs (31), and boosting the formation of portal vein tumor thrombi (PVTT) (29) and so on. Conversely, new data suggested that high Foxp3+ T cells in HCC improved the clinical outcome by suppressing proliferation, migration, and invasion of tumor cells (33) (**Figure 1**). Gong and colleagues first reported that those with high Foxp3 protein expression had better overall survival than those with low Foxp3 expression in 84 HCC patients. Based on those finds, they demonstrated that Foxp3 suppressed tumor growth in mouse tumor models by directly or indirectly inhibiting oncogene c-Myc (33).

In conclusion, most studies have found that a high frequency of infiltrating Foxp3+ T cells was associated with a poor prognosis in HCC. However, further study is needed to reach a consensus and explore the functions of nTregs and iTregs.

## CD8+ T Lymphocytes

Cytotoxic T lymphocytes (CTLs) always have CD8 surface antigen and play a function in the antitumor immune response. In current studies, CD8+ T lymphocyte is the most prevalent T cell subset. It has been shown that high expression of CD8+ TILs linked to a good prognosis in multiple tumors, including colorectal cancer (19), esophageal cancer (34), gastric cancer (35). In HCC, CD8+ T cells are also widely concerned. Several recent investigations have stated that CD8+ TILs expression decreased in the tumor tissue with HCC. Based on immunohistochemistry staining for CD8+ TILs from 29 cases with HCC, Yarchoan’s team found that the expression of CD8+T cells in the tumor tissue was remarkably lower than that in the liver background, and the expression of CD8+ T cells in the tumor side was lower than that in the nontumor side of tumor interface (36). Furthermore, the distribution of CD8+ T lymphocytes in HCC significantly varied among different areas and etiologies of the tumor. In non-alcoholic steatohepatitis (NASH) patients and animal models, CD8+ T cells were accumulated with strong programmed cell death protein 1 (PD-1) expression (37). Compared to the invasive margin region of the tumor in HCC, the central tumor region had a lower expression and a smaller area of CD8+ T lymphocytes (38). This phenomenon was also considered to happen because CD8+ T lymphocytes usually migrated from the peritumor region towards the central region of the tumor, and Tregs contributed to suppressing their migration and proliferation (32). While due to the perspective that HCC is a solid human tumor with abundant neovascularization and high perivascular infiltration of CD8+ T cells, the infiltration of immune cells was also believed to originate from intratumor vessel extravasation, not the peritumor tissue migration (39).

As in other tumors, many experts have demonstrated that CD8+ T lymphocyte in HCC was a protective factor (38–42) (**Figure 1**). According to research, high expression of CD8+TILs in the invasive margin, intratumoral, and perivascular regions has been linked to improved survival (39, 41). In a cohort analysis of 446 HCC cases, the densities of CD8+ TILs both in the intratumor and margin area positively correlated with overall survival and disease-free survival, and a higher density CD8+ TILs indicated a reduced recurrence rate (38). In addition, Sideras et al. reported that patients with a low density of CD8+ TILs survived poorly after analyzing stored formalin-fixed paraffin-embedded HCC tissue samples from 154 patients using immunohistochemical analysis, which was also confirmed in their validation cohort (42). This result suggested that CD8+ TILs with high expression had a positive prognostic role. Yet, whether CD8+ TILs could exert antitumor activity normally was still determined by the expression level of inhibitory receptors on their surface. Another study on NASH-related HCC discovered that the elimination of enriched CD8+ PD-1 T cells, which represent exhausted effector cells, could reduce liver damage and HCC incidence. In comparison to virus-related HCC, NASH-related HCC responded to immunotherapy treatment worse due to the obviously increased expression of CD8+ PD-1 T cells (37). Interestingly, a few studies came to an opposite view that high infiltrating CD8+ T lymphocytes predicted high recurrence and poor prognosis (28, 43, 44), which contradicted the antitumor role of CTLs.

Overall, CD8+TIL acting as an antitumor effector decreased in the tumor tissue, which has been proved to link with poor prognosis in HCC in the majority of studies. In NASH-related HCC, however, accumulating CD8+ T cells with elevated PD-1 expression accelerated tumor growth and reduced immunotherapy response.

## CD3+ T Lymphocytes

CD3 also is the typical surface antigen of T lymphocytes. Compared with HCV-associated cirrhosis, CD3+ TILs expression in HCV-associated HCC was significantly higher (43). In addition, the CD3+ TILs density of the intratumoral region was higher than that of the peritumoral area (45). Now the role of CD3+ TILs in HCC remains uncertain. Most studies showed that high CD3+ TILs expression was a protective factor against HCC (12, 38, 39, 46) (**Figure 1**). Patients having more CD3+ TILs in the intratumor tissue lived longer in HCC, according to Sun’s findings. This result, however, was not seen in the peritumor tissue. They came to the conclusion that CD3+ TILs in the central zone tumor was an independent indicator for HCC prognosis while the distribution of CD3^+^ TILs in peritumor had little impact on the prognostic value (38). In another research of 65 patients with I to IV stage HCC, high CD3+ TILs densities both in the center and margin of the tumors were found to predict low recurrence and prolonged disease-free survival (46). Yao’s group also came to the same conclusion in a meta-analysis that included 23 relevant papers with 3173 HCC patients (12). The value of CD3+ TILs in the perivascular area was likewise proved. A recent study found that patients with a high amount of CD3+ TILs at the perivascular region had long disease-free survival, implying that CD3+ TILs in the perivascular location could also be used as an independent predictor of HCC (39). Despite the above, the following paper showed that high CD3+ TILs was a poor prognostic factor for HCC (41). A meta-analysis of seven articles on TILs involving 1274 HCC patients revealed that high infiltration of CD3+ T lymphocytes at the tumor center was correlated with poor overall survival (**Figure 1**), whereas there was no correlation between CD3+ TILs density at the margin tumor and overall survival (41).

In summary, even though there were disputes, most studies found that a high concentration of CD3+ TILs in the tumor or perivascular region predicted a favorable outcome in HCC.

## CD4+ T Lymphocytes

CD4+ T lymphocytes took on activities in the immune response against tumor by secreting cytokines and activating CD8+ T lymphocytes (31, 47). Naive CD4+ T cells can differentiate into many subsets following antigens stimulation, including T helper 1 (Th1), Th2, Th9, Th17, Th22, follicular helper T (Tfh) cells, and Tregs. In recent years, CD4+ T cells with cytotoxic activity, which produce granzyme A, granzyme B, and perforin, have been termed CD4+ CTLs. It has been shown that CD4+ CTLs could exert antiviral and antitumor effects (48). CD4+ T cells have been extensively discovered in many cancers including HCC. It has been found that the numbers of circulating CD4+T lymphocytes were significantly higher in HBV-associated HCC patients than that in chronic hepatitis B (CHB), HBV-related cirrhosis patients, and healthy individuals (31). Furthermore, HCV-related HCC had higher CD4+ TILs than HCV-related cirrhosis (43). When patients progressed to HCC, CD4+ T lymphocytes were redistributed in tumor tissue, with CD4+ TILs expression in the peritumoral area being greater than that in the intratumoral area (28). CD4+ TILs might be decreasing as HCC progressed. Compared with the early-stage HCC, the quantity of CD4+ TILs was lower in the advanced-stage HCC, suggesting that the gradual reduction of CD4+ TILs might correlate with HCC progression (31). In order to better understand the mechanism of CD4+ TILs decline in late-stage HCC, Chaoul’s group observed the subsets of CD4+ TILs. When compared to the circulating blood of HCC patients, they found that terminally differentiated effectors (TEFF) strongly reduced and exhausted in both peritumor and central tumor, indicating that CD4+ TILs were selectively recruited into the tumor to escape the immune system (49).

It was found that CD4+ TILs served as a protective factor in HCC (31, 50) (**Figure 1**). The progressive decrease of CD4+ CTLs was related to a poor prognosis and a high recurrence of HCC (31). Another study performed in the NASH mice model revealed that inducing apoptosis of CD4+ T lymphocytes could promote HCC development while rescuing apoptosis of CD4+ T lymphocytes could prevent HCC development (50). Nevertheless, the mechanism of HCC progression mediated by a reduction of CD4+ TILs remains unclear. On the one hand, it was directly responsible for the reduced and exhausted TEFF of CD4+ TILs. On the other hand, insufficient stimulation of CD8+ TILs without the assistance of CD4 +TILs might play a potent role in the poor prognosis of HCC (51). However, several researches argued that there was no correlation between CD4+ TILs and HCC progression (12, 45). Even though positively correlated with elevated AFP and poor tumor differentiation, CD4+ TILs were discovered to be associated with neither overall survival nor disease-free survival (45).

Collectively, the decreased and impaired CD4+ TILs were observed in tumor tissue, which triggered the impaired activation of CD8+ TILs, and correlated with poor prognosis in HCC.

## Foxp3+/CD3+, Foxp3+/CD4+, Foxp3+/CD8+, CD8+/CD3+, CD4+/CD8+T Lymphocytes Ratio

As stated previously, tumor-infiltrating T lymphocytes with multiple subgroups exhibited antitumor and protumor effects at the same time, showing significant predictive value for HCC. So it is necessary to discover their prognostic value of the different ratios between TIL subsets for HCC. Low Foxp3+/CD3+, Foxp3+/CD4+, Foxp3+/CD8+TIL ratio, and high CD4+/CD8 +TIL ratio were shown to correlate with good prognosis (**Figure 1**), while CD8+/CD3+TIL ratio showed no correlation with survival in HCC (12, 52–54). Yao and co-workers reported that patients with lower Foxp3+/CD4+ and Foxp3+/CD8+TIL ratios had better overall survival and disease-free survival (12). A recent study found that the mortality rate of the high Foxp3 +/CD4+ TIL ratio group was 3.5 times higher than that of the low ratio group after observing the distribution of immune cells of 57 HCC patients in tumor tissue and peritumor tissue and the correlation of immune cells with clinical outcome. Then it came to a conclusion that the Foxp3+/CD4+ TIL ratio in tumor tissue was an independent prognostic factor for HCC (54). Additionally, Mathai’s group discovered that a high Foxp3 +/CD8+TIL ratio was correlated with poor tumor differentiation, high recurrence, poor overall survival, and disease-free survival in post-surgery HCC patients (52). Finally, in 2006, a report including 69 HCC patients who accepted liver transplantation from 1998 to 2001 pointed out that patients with a high CD4 +/CD8+TIL ratio had low recurrence risk after treatment (53).

Given the above, the ratio between TIL subtypes might be a useful prognostic indicator in HCC. Low Foxp3+/CD3+, Foxp3+/CD4+, and Foxp3+/CD8+TIL ratio, as well as high CD4+/CD8+TIL ratio were found to predict a good prognosis in recent studies.

## Interactions Between Tumor-Infiltrating T Lymphocytes and Other Immune Cells in HCC

### B Lymphocytes

B cells infiltrating in tumor tissue are defined as tumor-infiltrating B cells (TIBs). Under the stimulation of different factors and cells in TME, TIBs can differentiate into different subtypes and then play a dual role in tumors by secreting antibodies, acting as antigen-presenting cells (APCs), and secreting cytokines (55). TIBs have been widely focused on many different types of tumors, such as liver cancer (56), breast cancer (57), oropharyngeal squamous cell carcinoma (58), cervical cancer (59). High levels of TIBs, which have been proved to be a protective factor against HCC, were linked to smaller tumors and the lack of vascular invasion (60) (**Figure 1**). Multiple subsets of TIBs, including CD20+ B cells, naive B cells (Bn), IgM+ memory B cells (Bm), CD27− isotype-switched memory B cells (CD27− Sw Bm), as well as plasma cells (PCs), co-existed in HCC. In HCC tumors, all five subtypes were reduced and impaired as compared to non-tumor tissue. High Bn and CD27− Sw Bm densities might be utilized as independent good predictor factors, possibly because of their cytokines secreting activity, such as interferon (IFN)- γ (61). Furthermore, B cells might boost the humoral response to eliminate tumor cells by secreting antitumor antibodies (62). Last, it has been shown that TIBs were able to influence HCC progression through interacting with other immune cells (63). TIBs could increase the infiltration of CD8+ T cells *via* IFN-γ, IL-12p40, granzyme B to inhibit HCC progression (60) (**Figure 1**). TIBs secreting IL-12 and IFN-γ also stimulated NK cells, which had an anticancer impact (64) (**Figure 1**). Regulating B cells (Bregs), a novel subset of B cells, could promote HCC by interacting with many immune cells. When compared to healthy liver tissue, the amount of Bregs was elevated while the level of CD4+ CTLs expressing granzyme and perforin was downregulated in HBV tumor tissue, according to Xue’s group (65) (**Figure 1**). Finally, they concluded that the interactions between Bregs and CD4+CTLs might be the mechanism for HCC progression. Bregs also promoted HCC by blocking CD8+ T cells and NK cells while increasing Foxp3+ T cells (66) (**Figure 1**).

The roles of TIBs, as outlined, are still open for debate in HCC. The dual roles of TIB were determined by its subtypes infiltrating in HCC.

### Macrophages

There are two prominent macrophages in liver tissue: tissue-resident macrophages, such as Kupffer cells, and monocyte-derived macrophages (MDMs) (67). MDMs could differentiate into two different functional subtypes in TME, including M1 induced by lipopolysaccharide (LPS), IFN-γ, and M2 or tumor-associated macrophages (TAMs) induced by IL-4 and IL-10 (68). M1 macrophages mainly exert pro-inflammatory function, while M2 macrophages have an anti-inflammatory effect (69). Meanwhile, recent evidence suggested that M2 macrophages contributed to the progression of liver cancer by promoting pathogenic angiogenesis (70), tumor cell invasion and migration (71), epithelial-mesenchymal transition (EMT), and cancer stem cell-like characters (72, 73), as well as mediating drug resistance in HCC (74). According to research published in 2019 (74), not only did M2 macrophages prompt the progression and metastasis of liver cancer but also maintained the growth and metastasis of tumor cells by secreting hepatocyte growth factor (HGF), which significantly enhanced the resistance to sorafenib in liver cancer. Moreover, it was proposed that the interactions between macrophages and infiltrating T lymphocytes also contributed to liver cancer progression (**Figure 1**). Kuang et al. (75) reported that the distribution of CD68+TAMs in liver cancer tissue was positively correlated with that of Th17, and TAMs could induce the expansion of Th17, playing a role in fostering angiogenesis of cancer through secreting inflammatory factors, which promoted liver cancer finally. In addition, several studies have demonstrated that Tregs in tumor tissue were increased and activated by intratumoral macrophages, and their interactions promoted HCC progression at last (76, 77).

Finally, macrophages were proved to aid HCC progression through interacting with infiltrating T lymphocytes, such as Th17 and Tregs.

### Dendritic Cells

In the healthy liver, DCs mainly captured antigens as APCs, presented antigens to T cells with the help of major histocompatibility complex (MHC), and then activated antigen-specific T lymphocytes, which linked innate and adaptive immunity together. The precise role of infiltrating DCs in HCC remains uncertain. It was widely thought that high infiltration of DCs was associated with a favorable prognosis and could predict recurrence and metastasis independently in individuals following surgery (78) (**Figure 1**). Their antitumor function was generated due to the mechanism that DCs could activate T cells responses to inhibit HCC progression (79). Other studies, on the other hand, revealed that DCs could be suppressed, leading to the development of HCC in patients with high AFP levels (80). Cross-talks between DCs and other cells, such as tumor cells and immune cells, have also been discussed in some studies. It has been shown that tumor cells could induce the immature differentiation of DCs by reducing adhesion molecules or secreting immune inhibition cytokines, such as IL-10 and vascular endothelial growth factor (VEGF) (81, 82). Compared with mature DCs, immature DCs could recognize and process antigens well while present antigens poorly (83). As a result, DC-T cell interactions were reduced, and T cells could not fully play the role of antitumor effector cells, which might be one of the mechanisms of immune resistance to tumors (84) (**Figure 1**). In an ex vivo study, DCs with low IL-12 production caused the functional impairment of T lymphocytes, confirming the interaction between them (85). In addition, another recent evidence (86) confirmed that the expression of inhibitory receptors on the surface of CD4+ and CD8+ T cells, such as PD-1, T-cell immunoglobulin and mucin-domain containing-3 (TIM3), cytotoxic T-lymphocyte-associated protein 4 (CTLA4), was higher in the tumor tissue than that in the non-tumor tissue and peripheral blood. Those inhibitory receptors could combine with corresponding inhibitory ligands of DCs and tumor cells, induce the disability of CD4+ TILs or suppress the cytotoxic reaction of CD8+ TILs, which finally mediated the immune tolerance to the tumor (**Figure 1**). The latest immune check-point treatment was founded on those theory bases and had been used in many types of tumors, including liver cancer. Compared with traditional tumor therapies, immune checkpoint treatment could recover the response of impaired TILs to tumor antigens and prolong the survival of HCC (84, 86).

As can be observed, DCs had a role in the development of HCC due to inadequate DC-T interactions and an increased inhibitory function on effector T cells.

### Natural Killer Cells

NK cells, which belong to innate immune cells, account for 5-20% of circulating lymphocytes and over 50% of intrahepatic lymphocytes. They mainly recognize abnormal cells with low expression of MHC I, such as tumor cells and infected cells. Then, activating receptors on NK cells will be activated, which causes an unbalance towards activated NK cells, and NK cells can eradicate target cells directly or indirectly, such as NK cell-mediated cytotoxicity and secreting pro-inflammatory cytokines (87) (**Figure 1**). Compared to healthy liver tissue, the quantity of infiltrating NK cells changed, and their function did in HCC tissue. Wu’s group (88) reported that the number of infiltrating NK cells in HCC tissue significantly reduced, and their functions were also impaired, which might trigger immune evasion of the tumor and finally cause the progression of HCC. This suggested that NK cells had the antitumor role. Consistent with that, another study discovered that higher frequency of NK cells of intratumor tissues was related to longer recurrence-free and overall survival in HCC patients treated with sorafinib (89). Aside from the reduced expression and dysfunction of NKs, the following mechanisms involved in NKs might promote HCC progression. First, the final result of HCC patients was determined by the balance between infiltrating NKs, which could destroy tumors, and other immune cells, such as Tregs, which might inhibit immune response. Wang and co-workers (90) found that, compared with non-tumor tissue, both Tregs and the activated NK cells significantly increased in liver cancer tissue. Now, the increased Tregs were shown to be associated with poor prognosis in HCC (91). Second, the state of NK cells would be influenced by tumor cells. On the surface of NK cells, there were activating receptors that could activate NKs to exert immunity surveillance and inhibitory receptors which could inhibit NKs to trigger immune evasion, and their imbalance would determine the function of NKs in HCC (92). NKG2D was one of the most well discussed activating receptors in HCC. Although, tumor cells might still manage to escape the immune surveillance by down-regulating NKG2D *via* increased TGF-β secretion (93) or decreased expression of its ligands, such as major histocompatibility chain-related protein A (MICA) (94). Third, NKs might promote HCC progression *via* interacting with infiltrating T cells. An earlier review (95) discovered that antigen-specify T lymphocytes regulated by NK cells would boost as the depletion of NK cells. Hence, it concluded that NK cells finally promoted HCC progression by lowering the immune activity against tumors *via* diminishing T cells (**Figure 1**). It has been found that Treg cells could directly inhibit the function of NK cells by expressing membrane-bound TGF-β (96), or reduce the sensitivity of NK cells to tumor cells *via* diminishing IL-2 (95) (**Figure 1**).

Ultimately, NKs failed to exert the antitumor effect for the sake of their frequency decreasing, function impairment, and interaction with surrounding tumor cells or other types of infiltrating immune cells in HCC.

## Critical Signaling Pathways Related to TILs in HCC

### TGF-β Signaling

**Figure 2:**
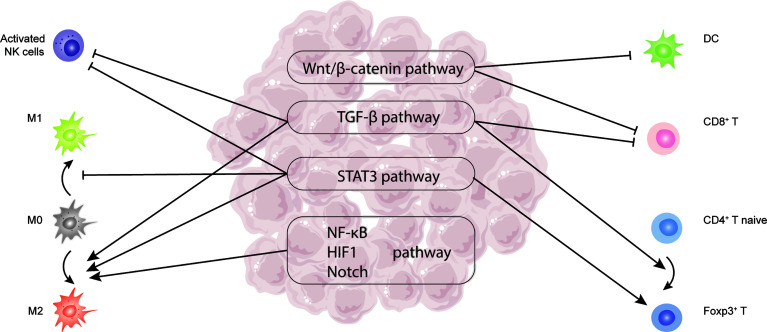
Critical signaling pathways related to TILs in HCC, including TGF-β, STAT3, Wnt/β-catenin, NF-κB, HIF-1, and Notch pathway.

The TGF-β family comprises TGF-βs, activins, inhibins, bone morphogenetic proteins (BMPs), as well as growth and differentiation factors (GDFs). The activation of this signaling pathway starts with the binding of ligands to the extracellular region of TGF-β type I and type II receptors (TβRI and TβRII), followed by activating SMAD-dependent and SMAD-independent pathways to trigger downstream cascades. In HCC, TGF-β signaling is involved in almost each stage of tumor formation (97). TME had a large amount of TGF-β, which was overexpressed by tumor cells and other immune cells. During the early stage of liver cancer, TGF-β suppressed the proliferation of premalignant hepatocytes. But in the advanced HCC phase, it contributed to tumor progression through regulating immune cells, such as Tregs, CTLs, TAMs, NKs (98). First, TGF-β could induce the expression of Foxp3 on CD4+CD25- naive T cells through activating SMAD-dependent pathways, mediating the formation of Tregs (99) (**Figure 2**). Tregs in periphery blood also infiltrated into HCC tumor tissues to suppress the antitumor effect of CTLs (100) (**Figure 2**). In addition, tumor cells of HCC increasingly expressed TGF-β, which was capable of upregulating the expression of PD-1 on CD8+ CTLs. CTLs then bound to the PD-L1 on tumor cells and APC to induce CTLs exhaustion (101). At last, TGF-β signaling could participate in the regulation of the innate immune system by directly suppressing NK cells and promoting the differentiation of TAM towards M2 to induce immune escape (102) (**Figure 2**). Taken together, TGF-β signaling pathways were broadly involved in the regulation of TILs, and finally exerted promotion function in HCC.

### Signal Transducer and Activator of Transcription 3 Signaling

The human signal transducer and activator of transcription (STAT) protein family consist of 7 members, including STAT1, STAT2, STAT3, STAT4, STAT5A, STAT5B, and STAT6. Among them, STAT3 has been focused by many studies on cancer regulation. STAT3 was thought to be activated rapidly and transiently in healthy cells by several cytokines, such as IL-6, IL-10, IFNs, and growth factors, such as VEGF. Nevertheless, it was stimulated persistently and abnormally in tumor cells. According to a recent study, phosphorylated STAT3 could be detected in around 60% of liver cancer samples, which was related to poor prognosis (103). STAT3 signaling pathway mainly exerted protumor function through regulating DCs, differentiation of TAMs, NKs, and Tregs. In DCs, IL-6 secreted by liver tumor cells and hepatic carcinoma-associated fibroblasts (hCAFs) could bind to the corresponding IL-6R on DCs, activate janus kinase (JAK), and then start the downstream phosphorylation cascade of STAT3 signaling pathway to inhibit T cells proliferation and increase Tregs production (104) (**Figure 2**). Moreover, STAT3 signaling was proved to inhibit the expression of activation receptor NKD2G on NKs and its corresponding ligands MICA/B on tumor cells, which could block the activation of NKs and result in failing immune surveillance in HCC (**Figure 2**). Suppressing STAT3 signaling in HCC could reactive NKs to exert antitumor function through altering cytokines in TME, such as reducing the level of IL-10 (105). Apart from that, IL‐6/STAT3 signaling facilitated tumor development by reducing M1 polarization while increasing M2 differentiation of TAM (106) (**Figure 2**). As can be seen, STAT3 signaling influenced multiple immune cells, which aided in the development of HCC.

### Wnt/β-Catenin Signaling

When Wnt ligands bind to their corresponding receptors on the surface of cells, the Wnt signaling cascade is activated. β-catenin, usually located in the adherent junctions and cytoplasm of cells, is driven to accumulate in the cytoplasm and translocate into the nucleus, where it exerts regulation function (107). Wnt/β-catenin signaling was frequently stimulated in HCC (108), which mainly reduced the frequency of TILs and impaired their function (109). The activation of Wnt/-catenin signaling in HCC was considered to impair the innate immune system by lowering DC infiltration (**Figure 2**), which then impaired the adaptive immune response through reducing antigen-specific CD8+ T cell migration (110). Apart from that, Wnt/-catenin signaling also interfered with the effect of CTLs, causing them to become exhausted (111) (**Figure 2**). In summary, Wnt/β-catenin signaling was frequently activated and promoted HCC through interacting with TILs, such as CD8+ T cells and DCs.

### Other Signaling Pathways

NF-κB signaling pathway including canonical and non-canonical plays significant roles in many kinds of liver disease, such as hepatitis, liver cirrhosis, and liver cancer (112). In HCC, hepatoma-derived factors expressed by tumor cells could bind to hepatoma-derived toll-like receptor 2 (TLR2) to active canonical NF-κB signaling pathway. Subsequently, TAMs differentiation towards M2 was enhanced and acted as protumor function (113) (**Figure 2**). Moreover, another two signaling pathways, named HIF-1 and Notch signaling, have been proved to be involved in the recruitment and activation of TAM in HCC (114, 115) (**Figure 2**). Ultimately, the formation and development of HCC are complicated. The signaling pathways related to TILs in HCC played potent roles in this process. And further research is still needed to clarify the mechanisms of HCC progression deeply.

## Conclusion

Overall, TILs have been shown to exhibit both antitumor and protumor properties in HCC. They cross-talked to create a complex map with multiple signaling pathways connecting them in the development of HCC. Future investigations are necessary to reveal the undiscovered TIL domains, such as the specific functions and mechanisms through which TILs function. In the long term, studying on TILs in the roles and mechanisms of HCC progression might help us best understand the law of HCC progression, and identify innovative immunotherapy strategies.

## Author Contributions

XZ prepared and wrote the manuscript. WJ drafted and corrected the figures. SW contributed to design the manuscript of the second edition. HD critically revised and edited the manuscript content. All authors contributed to the article and approved the submitted version.

## Funding

This study was supported by National Natural Science Foundation (81672725) and Sino-German Cooperation Group (GZ1517).

## Conflict of Interest

The authors declare that the research was conducted in the absence of any commercial or financial relationships that could be construed as a potential conflict of interest.

## Publisher’s Note

All claims expressed in this article are solely those of the authors and do not necessarily represent those of their affiliated organizations, or those of the publisher, the editors and the reviewers. Any product that may be evaluated in this article, or claim that may be made by its manufacturer, is not guaranteed or endorsed by the publisher.
